# Evaluation of periodontal risk assessment model among adults aged 30-60 years attending KLE Dental College, Belgaum: A hospital-based study

**DOI:** 10.4103/0972-124X.75912

**Published:** 2010

**Authors:** Shruthi Eshwar, Anil V. Ankola, Ashok Kumar, Mamata Hebbal

**Affiliations:** *Department of Preventive and Community Dentistry, K.L.E.V.K. Institute of Dental Sciences, Belgaum, Karnataka, India*

**Keywords:** Computerized model, periodontal risk, risk assessment, risk factors

## Abstract

**Aim:**

The aim of the present study was to evaluate the periodontal risk of individuals using the modified periodontal risk assessment model.

**Materials and Methods:**

Adult subjects aged 30-60 years attending the out patient department of Institute of Dental Sciences, Belgaum in a week’s period were screened and 30 among those who met the criteria were included in the study. Complete history and examination of the oral cavity was done using mouth mirror and community periodontal index probes. Periodontal status was recorded using community periodontal index. Systemic conditions like hypertension and diabetes was assessed by suitable investigations. All the risk factors were plotted on a model using Microsoft excel and periodontal risk was assessed based on the findings and categorized as low, moderate and high risk.

**Results:**

Among 30 patients 13 were in low risk group, 10 in moderate risk group, and 7 in high risk group identified by proposed model given by Vishwa Chandra whereas 20 patients were in low risk group, 5 in moderate risk group and 5 in high risk group when identified Lang and Tonetti model (2003).

**Conclusion:**

In conclusion the use of risk assessment tool would result in reduction of complex therapies and would prevent the future effects of periodontal disease such as bone and tooth loss.

## INTRODUCTION

Dental caries and periodontal disease are the major causes of tooth loss. Until around the 1970s, virtually all children and adults in the United States had dental caries, and almost all adults developed periodontal disease. In the 1950s, approximately 80 % of children 13 to 15 years of age had gingivitis. Periodontitis was observed to begin in the late teenage years and to increase almost linearly until early middle age, after which close to 100% of the adult population below the age of 60 years was affected. Tooth loss began in the late teenage years and increased linearly through age 60. Virtually all children and adults also manifested dental caries. Both diseases were ubiquitous throughout the dentulous population.[1]

Traditionally, management of both diseases has been based onthe repair model of care under which the clinician’s goalwas to diagnose the problems and resolve them via treatment. Treatments were empirical and basically the same for all patients. The concepts that host factors are important in the pathophysiologyof periodontitis and that individuals may vary greatly in theirlevel of risk had not been conceived until the 1970s. Preventivemeasures were largely ignored; and later, when they were used, they were not applied uniformly throughout the population.[2]

Knowledge about the nature of caries and periodontal diseasehas increased enormously since the 1970s, resulting in changingdiagnostic and treatment paradigms. The evidence demonstratesthat although periodontal disease and caries are infectious, bacteria alone are insufficient. A susceptible host is alsoessential for disease to occur. Susceptibility and risk fordisease vary greatly from one individual to another, and majorfactors that place individuals at risk have been identified.[3]

The assessment of risk caused by periodontal disease is an essential factor during treatment and maintenance phases. In trying to make a comprehensive assessment of risk caused by periodontal diseases, several factors need to be considered and analyzed, leading to a great degree of variation in the assessment of risk between general dentists and periodontists and between periodontists themselves. Manually summarizing and analyzing these factors could be a complex process, and a computer generated risk assessment model becomes a necessity to assess the risk that arises from various forms of periodontal disease.[4] Various risk assessment models for periodontal disease are in vogue, such as the Oral Health Information Suite (OHIS) and the Periodontal Risk Calculator (PRC). In addition, these models have been used prospectively or retrospectively to assess risk, thus confounding periodontists both in the selection as well as in the interpretation of data from a risk assessment model.[5]

In the era where there is a transition occurring in periodontics from a normal health care delivery model to a complete wellness model, there is now an attempt to create and develop tools which can aid a general dentist, periodontist or even a patient to assess his/ her oral hygiene and periodontal status.

Thus the aim of the present study was to evaluate the periodontal risk of individuals using modified periodontal risk assessment (PRA) model

## AIM AND OBJECTIVES

### Aim

To evaluate the periodontal risk of individuals, using modified periodontal risk assessment (PRA) model.

### Objectives

To obtain data regarding different factors influencing periodontal health.

To compare the model with the old model (Lang and Tonetti model, 2003).

To classify individuals according to their different risk levels based on the models.

## MATERIALS AND METHODS

This was a descriptive crosssectional study.

### Inclusion criteria

1. Patients who gave informed consent
2. Patients aged between 30 and 60 years

### Exclusion criteria

1. Patients with difficulty in mouth-opening
2. Patients with less than 20 functional teeth

### Method of collection of data

Adult subjects aged 30 to 60 years attending the outpatient department of the Institute of Dental Sciences in a week’s period were screened, and 30 among those who met the inclusion and exclusion criteria were included in the study. Informed consent was taken before the start of the study, and ethical clearance was obtained from the committee. Complete history and examination of the oral cavity was done using mouth mirror and community periodontal index (CPI) probes. Periodontal status was assessed by parameters like bleeding on probing, periodontal pockets, loss of attachment determined by using CPI.

Systemic conditions like hypertension or diabetes were investigated by suitable tests.

Socioeconomic status of the subjects was determined using Kuppuswamy classification.

All the risk factors were plotted on a PRA model using MicrosoftExcel, and the periodontal risk was assessed based on the findings and categorized as low, moderate and high.

### Subject risk assessment

The patient’s risk assessment for recurrence of periodontitis may be evaluated on the basis of a number of clinical conditions whereby no single parameter displays a more paramount role. The entire spectrum of risk factors and risk indicators ought to be evaluated simultaneously.[6] For this purpose, the risk assessment model described is an expansion of PRA model by Lang and Tonetti, 2003. It is a continuous multilevel risk assessment model that incorporates subjective tooth and site risk assessments and generates a functional diagram and depending on the area of polygon categorizes the patients into low-, moderate- and high-risk categories.[5] Four entities from the original model were retained in the new model: bleeding on probing, probing depth, tooth loss and smoking. The entities that were added included various aspects of risk assessment like diabetes, socioeconomic factors and stress. A comprehensive evaluation of the functional diagram will provide an individualized total risk profile.[5]

#### Bleeding on probing

Bleeding on probing is currently widely used as an indication that patient needs treatment. The absence of bleeding on three or more of the four occasions is an excellent indicator of health. Conversely, presence of bleeding on probing is not a predictor of future deterioration.[5]

#### Probing depth

Probing depth of more than 5 mm per se does not make much sense when considered as a sole parameter the evaluation in conjunction with other parameters will reflect existing ecological niches from which re-infection might occur. Pockets of more than 5 mm are assessed as the second indicator of risk for recurrent disease in the diagram.[6]

#### Loss of teeth

Although reason for tooth loss may not be known, the number of remaining teeth in dentition reflects the functionality of the dentition. Tooth loss also represents a true end point outcome variable reflecting patient’s history of oral diseases and trauma; it is logical to include this risk indicator as the third parameter.[6]

#### Attachment loss–to–age ratio

Loss of attachment and bone loss generally increase with aging, but pocket deepening may not correlate with these changes; thus the number of sites with >5 mm pocket depth may be important. Therefore, attachment loss to-age ratio was assessed as the fourth parameter.[5]

#### Diabetes as a parameter of risk indication

Susceptibility to periodontitis in patients with diabetes extends back to more than 60 years. The prevalence of periodontal disease is higher in both type 1 and type 2 diabetics. Patients with type 1 diabetes or type 2 diabetes do not differ with regard to risk when other variables are controlled. Individuals with uncontrolled diabetes are at risk of greater periodontitis.[5]

#### Smoking

Smoking affects the susceptibility of patients with chronic periodontitis. More recent evidence has established that smoking *per se* represents not only a risk marker but also probably a true risk factor for periodontitis. The association of smoking and periodontitis is dose dependent.[6]

#### Dental status–systemic factors interplay

Numerous other health problems may modify the progression, and the host response may vary between an inadequate response and an exaggerated response. Tooth risk factors such as iatrogenic factors, furcation involvement play a major role in accelerating periodontal disease. These may act as potential plaque-retentive areas which may be periodontopathic, especially if systemic disease is present.[5]

#### Background characteristics

Genetics, age, gender, stress and socioeconomic status are important background characteristics and also risk determinants. Economic status also pertains to decreased awareness of oral health and dental visits.

## RESULTS

In the present study 30 subjects aged between 30 -60 years were examined. Thorough examination & charting of periodontal status was carried out.

Table 1: Depicts the coding system of PRA model with various parameters like bleeding on probing, probing depth, tooth loss, attachment loss – age ratio, smoking and diabetic status with different axis scores.

**Table 1:** Coding system^5^

Axis score	Bop	Pd>5mm	Tooth loss	Smoking/Day	Al/Age	Diabetic status
0	0	0	0	Non smoker	0	<102
1	<4	1-2	1-2	Former smoker	<0.25	102-109
2	5-9	3-4	3-4	<10	0.26-0.5	110-117
3	10-16	5-6	5-6	10-19	0.51-0.75	118-125
4	17-25	7-8	7-8	20	0.76-1	126-133
5	>25	>9	>9	>20	>1	>133

Table 2: Explains the coding system for dental status & systemic factors interplay with various axis score ranging from 0–5 with healthy status to tooth morbidity.

**Table 2:** Coding systems for dental status – Systemic factors interplay^5^

Axis score	Status
0	Healthy
1	Healthy with minor problems not affecting periodontal status
2	Dental health problems affecting periodontal status(iatrogenic, endodontic)
3	General problems which modify periodontal status (genetic, nutrition, endocrine, psychosomator)
4	Severe dental problems
5	More severe with tooth morbidity

Table 3: evaluation of PRA model into various categories like low-, moderate- and high- risk.

**Table 3:** Evaluation of pra model^5^

Low risk	Moderate risk	High risk
All parameters in low risk areas, or at most 2 parameters in moderate and high risk area	3 in moderate one in high risk	2 parameters in high risk category

**Figure 1:**
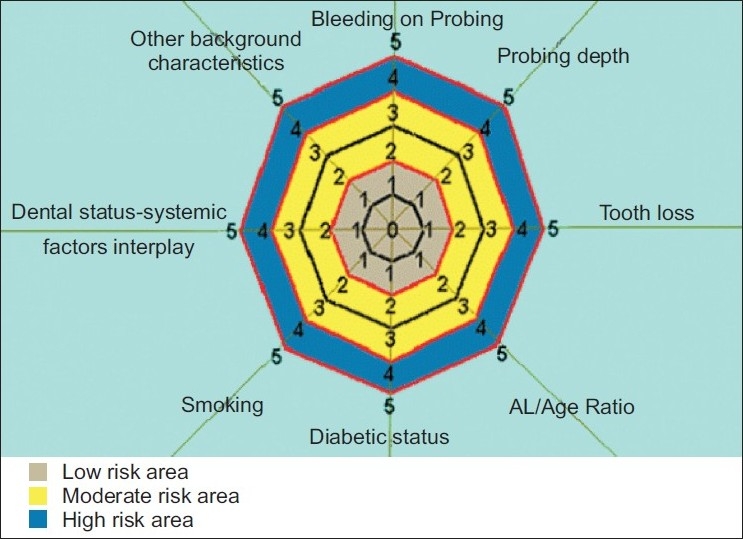
This figure depicts the proposed model, which considers the cumulative periodontal status, risk factors and risk determinants under eight parameters and with clearly demarcated low-, moderate- and high- risk zones

Figure 1: PRA model - This figure depicts the proposed model, which considers the cumulative periodontal status, risk factors and risk determinants under eight parameters and with clearly demarcated low, moderate and high risk zones.

**Figure 2:**
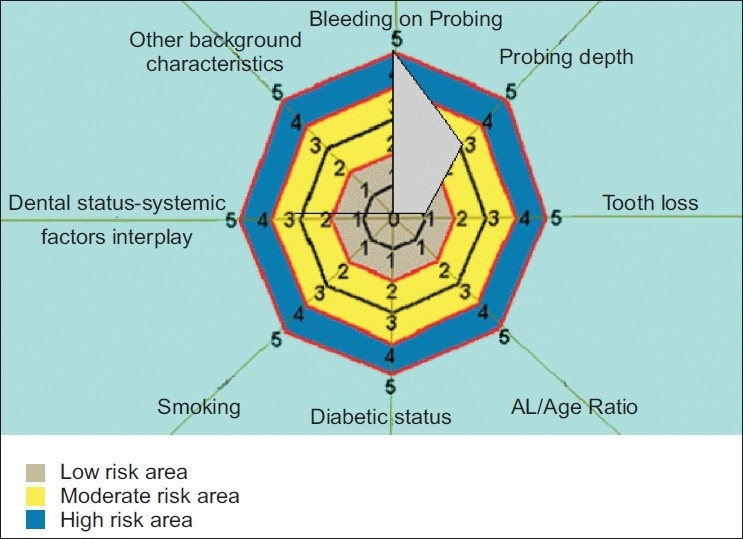
Risk diagram of low periodontal risk patient with generalized bleeding on probing and probing depth of 3-4 mm

Figure 2: Low Risk PRA model - Risk diagram of low periodontal risk patient with generalized bleeding on probing and probing depth of 3 -4 mm.

**Figure 3:**
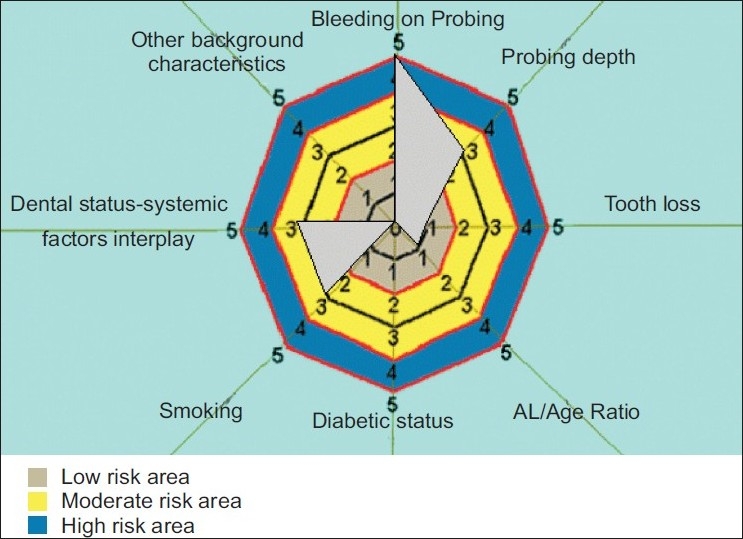
Risk diagram of moderate periodontal risk patient with generalized bleeding on probing and probing depth of more than 5 mm and with minor ental problems not affecting the periodontium, and smoking less than 10 cigar per day

Figure 3: Moderate risk PRA model - Risk diagram of moderate periodontal risk patient with generalized bleeding on probing and probing depth of more than 5 mm and with minor ental problems not affecting the periodontium, and smoking less than 10 cigar per day.

**Figure 4:**
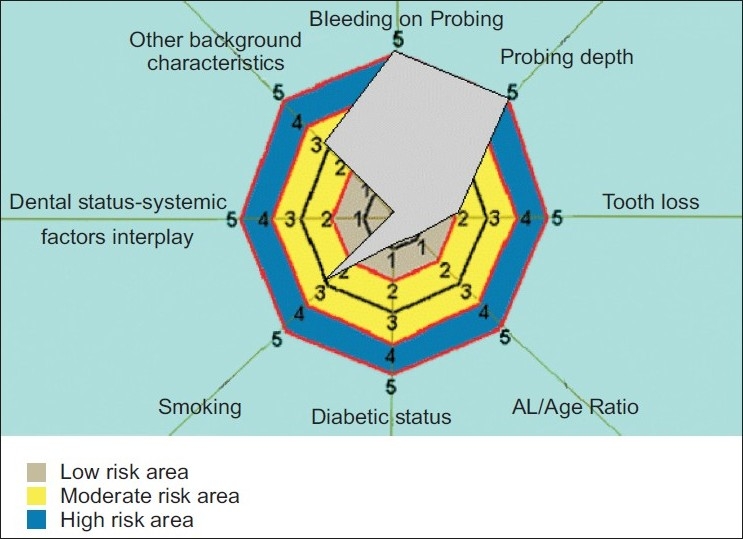
Risk diagram of high risk periodontal patient with generalized bleeding on probing, pocket depth of more than 8mm, and smoking 10– 19 cigar per day

Figure 4: High risk PRA model - Risk diagram of high risk periodontal patient with generalized bleeding on probing, pocket depth of more than 8mm, and smoking 10–19 cigar per day.

1. All patients had generalized bleeding on probing
2. 12 (46%) patients were smokers and 4 (15%) former smoker,
3. Patients were confirmed diabetics
4. 50% of the patients had periodontal pockets more than 5 mm at 7–8 areas and 35% had pockets of 5 mm at 5-8 areas and 47% had loss of attachment (3-5 mm) and only 10% had loss of attachment of more than 5mm
5. 35% of the subjects had 7-8 missing teeth
6. Most of the subjects belonged to lower middle class and lower class
7. Among 30 patients 13 were in low risk group, 10 in moderate risk group, and 7 in high risk group identified by proposed model given by Vishwa Chandra whereas 20 patients were in low risk group, 5 in moderate risk group and 5 in high risk group when identified Lang and Tonetti model (2003).

## DISCUSSION

The present study was aimed toward two different goals.

1. The first goal was to evaluate the PRA model and to compare the risk assessment capability of the proposed model with the original model.
2. To perform a limited test of applicability of this model in general dentistry.

The model used is both userfriendly and simple to use by everyday clinicians and at the same time advanced enough to be used as an aid for oral health management in general dental clinics. Various risk assessment models are developed before and are in vogue. They range from simple questionnaires to more complicated PRC (Page *et al*., 2003). The PRC evaluates nine risk parameters, namely, patient’s age, smoking, diagnosis of diabetes, history of periodontal surgery, probing depth, furcation involvement, calculus below gingival margin, radiographic bone height and presence of vertical bone lesions. Risk scores calculated using PRC during periodontal examination predict risk with high level of accuracy.[5]

Another risk assessment tool is oral health information suite (OHIS, Page *et al*., 2005). The OHIS is an information system that compiles, analyzes and quantifies clinical information about factors like current oral health status, interventions needed and treatment outcomes, be they beneficial or detrimental, that are attributable to treatment and behavioral decisions. The OHIS satisfies the need for a quantitative way to assess risk for periodontitis, as well as providing, for the first time, quantification of periodontal status and changes in status over time. This is very powerful information for all stakeholders. It provides patients with a superior understanding of their oral health status and the interventions recommended. The patient and clinician benefit from the objective measures of the outcomes and effectiveness of the interventions chosen. It permits payers of health care services to determine the value of health improvements achieved relative to the funds expended. Use of the OHIS enables a transition from the repair to the wellness model of dental care. The wellness model guides the clinician and patient toward a health care strategy based on risk reduction and disease prevention. Use of the wellness model over time may be expected to result in improved oral health, reduction in the need for complex therapy, and stabilization or reduction in oral health care costs.[3]

In the proposed model, all the parameters were evaluated on the scale of 1 to 5, with the scoring criteria loosely based on those of Renvert and Persson (2004) for the parameters in Lang and Tonetti’s model, in which all parameters were coded from 0 to 5 depending on clinical situation and findings. Regardless of the model used, it should be emphasized that the predictive value of most of the routine periodontal parameters is low. Bleeding on probing is highly significant, but higher levels cannot be used as a predictor of disease progression. However, deeper periodontal pockets are positively associated with progression of periodontitis; hence parameters like bleeding on probing and pocket depth must be interpreted with care and caution.[5]

The new model assessed in this study tries to incorporate both local and systemic factors, including dental factors that can initiate and modify the progression of periodontal disease and certain risk determinants. Local factors harbor periodontopathic bacteria, which if left undisrupted, can initiate periodontal disease.[7] These dental factorssystemic factors interplays tend to emphasize the importance of these factors, especially in presence of systemic disease that modifies normal mechanisms, which make up normal host response. Socioeconomic status also relates to decreased awareness and decreased dental visits.[8] There is an apparent association between psychosocial factors and risk behaviors such as smoking, poor oral hygiene and periodontitis. The original Lang and Tonetti model (2003) identified more of low risk cases, whereas the proposed model by Vishwa Chandra, which is based on PRA model, showed fewer cases in lowrisk group and almost equal in moderate- and high-risk groups. This can be attributed to two reasons: the higher number of parameters in the proposed model (6 and 8) and also to the criteria used for risk assessment. In the proposed model, for a patient to be identified as a moderate-periodontal risk patient, at least three parameters must be present; hence when this model was compared with the original model, there was a difference, with more patients falling into low-risk group in the old model.[5] Though every patient had all the factors which could influence periodontal disease, many were identified to be at low risk because the interaction between the 8 parameters was not so severe as to identify them as moderate- or high-risk patients. In the evaluation of the natural progression of periodontal disease, prospective studies are generally preferred over retrospective studies, and some risk assessment models are used that are effective in predicting the future periodontal status.[8] However, the new model was not assessed in this way, and the model is primarily a retrospective one, where information is gathered to assess the current risk for the patients; unlike other models, where current status is assessed and the future risk is predicted. The proposed model is easy to generate and obtain, and it uses retrospective and current data to assess the risk. It helps to sort out cost-effective treatments and even suggest rational approaches to preventive measures. It has the potential to substantially improve the health care by reducing over-treatment of low-risk patients and applying more aggressive preventive strategies to high-risk patients. In our view, sufficient knowledge now exists for clinical utilization of risk assessment models for periodontitis. Based on the information gathered, the practitioner can assess the degree of risk for the individual under consideration. Once established, the degree of risk becomes the integral part upon which a plan of treatment and maintenance is based.

To conclude, the new periodontal risk assessment model is described and compared to the model from which it is derived (PRA). The use of risk assessment tool overtime may be expected to result in more accurate clinical decision-making, as well as improved treatment protocols, and indirectly to result in reduction of complex therapies; and would prevent the future effects of periodontal disease such as bone and tooth loss.
